# Progranulin sustains STAT3 hyper‐activation and oncogenic function in colorectal cancer cells

**DOI:** 10.1002/1878-0261.12552

**Published:** 2019-08-10

**Authors:** Federica Laudisi, Fabio Cherubini, Antonio Di Grazia, Vincenzo Dinallo, Davide Di Fusco, Eleonora Franzè, Angela Ortenzi, Illari Salvatori, Silvia Scaricamazza, Ivan Monteleone, Naoya Sakamoto, Giovanni Monteleone, Carmine Stolfi

**Affiliations:** ^1^ Department of Systems Medicine University of ‘Tor Vergata’ Rome Italy; ^2^ IRCCS Fondazione Santa Lucia Rome Italy; ^3^ Department of Biology University of ‘Tor Vergata’ Rome Italy; ^4^ Department of Biomedicine and Prevention University of ‘Tor Vergata’ Rome Italy; ^5^ Department of Molecular Pathology Hiroshima University Hiroshima Japan

**Keywords:** anti‐apoptotic proteins, antisense oligonucleotide, cyclin D1, JAK‐STAT, SHP‐2, tumor‐infiltrating leukocytes

## Abstract

Persistent activation of Signal Transducer and Activator of Transcription (STAT)3 occurs in a high percentage of tumors, including colorectal cancer (CRC), thereby contributing to malignant cell proliferation and survival. Although STAT3 is recognized as an attractive therapeutic target in CRC, conventional approaches aimed at inhibiting its functions have met with several limitations. Moreover, the factors that sustain hyper‐activation of STAT3 in CRC are not yet fully understood. The identification of tumor‐specific STAT3 cofactors may facilitate the development of compounds that interfere exclusively with STAT3 activity in cancer cells. Here, we show that progranulin, a STAT3 cofactor, is upregulated in human CRC as compared to nontumor tissue/cells and its expression correlates with STAT3 activation. Progranulin physically interacts with STAT3 in CRC cells, and its knockdown with a specific antisense oligonucleotide (ASO) inhibits STAT3 activation and restrains the expression of STAT3‐related oncogenic proteins, thus causing cell cycle arrest and apoptosis. Moreover, progranulin knockdown reduces STAT3 phosphorylation and cell proliferation induced by tumor‐infiltrating leukocyte (TIL)‐derived supernatants in CRC cell lines and human CRC explants. These findings indicate that CRC exhibits overexpression of progranulin, and suggest a role for this protein in amplifying the STAT3 pathway in CRC.

**Abbreviations**
5‐FU: 5‐fluorouracil
ASO: antisense oligonucleotide
AV: Annexin V
BrdU: 5‐bromodeoxyuridine
CDK: cyclin‐dependent kinase
CPT‐11: camptothecin‐11
CRC: colorectal cancer
DAB: 3,3′‐diaminobenzidine
DMSO: dimethyl sulfoxide
DTT: dithiothreitol
EDTA: ethylenediaminetetraacetic acid
ERK: extracellular signal‐regulated kinase
FBS: fetal bovine serum
FITC: fluorescein isothiocyanate
HBSS: Hank's balanced salt solution
IL: interleukin
MAPK: mitogen‐activated protein kinase
PBS: phosphate‐buffered saline
PCR: polymerase chain reaction
PI: propidium iodide
siRNA: small interfering RNA
STAT3: signal transducer and activator of transcription 3
TIL: tumor‐infiltrating leukocyte
TNF: tumor necrosis factor

## 1 Introduction

Colorectal cancer (CRC) is the third most common form of malignancy and the second leading cause of cancer‐related mortality in the Western world (Center *et al*., 2009). CRC develops through a multistage process characterized by accumulation of aberrant protein expression/activation patterns, each granting a specific growth advantage to tumor cells (Fearon and Vogelstein, 1990). In recent years, increasing attention has been focused on transcription factors that lie at the hub of multiple oncogenic signaling pathways, such as Signal Transducer and Activator of Transcription (STAT)3 (Wake and Watson, 2015). Inappropriate/persistent STAT3 activation is described in a number of neoplasias, including CRC, and STAT3 blockade in cultured cancer cells inhibits cell proliferation and induces apoptosis (Xiong *et al*., 2014). Furthermore, STAT3 is considered as an attractive therapeutic target in CRC (Yu *et al*., 2014). However, conventional approaches aimed at inhibiting STAT3 functions have been challenged by some limitations (Beebe *et al*., 2018). For instance, due to the high similarity of STAT3 with STAT1, a STAT family member involved in cell death and defense against pathogens (Avalle *et al*., 2012), STAT3 inhibitors can enhance the risk of infections (Nero *et al*., 2014). Moreover, as STAT3 is required for the survival of normal intestinal epithelial cells and maintenance of mucosal integrity, persistent interference with STAT3 activation could potentially promote gastrointestinal damage (Grivennikov *et al*., 2009).

Because STAT3 mediates distinct biological effects by interacting with specific cooperating proteins (Laudisi *et al*., 2018), the identification of tumor‐specific STAT3 cofactors could facilitate the development of compounds that interfere exclusively with STAT3 in cancer cells. Progranulin, also known as granulin/epithelin precursor, acrogranin, and proepithelin, is a pleiotropic growth factor playing a role in the maintenance and regulation of the homeostatic mechanisms of proliferation, normal tissue development, regeneration, and the host‐defense response (Jian *et al*., 2013). Progranulin overexpression occurs in different types of cancer and associates with a poor prognosis (Arechavaleta‐Velasco *et al*., 2017). Progranulin acts as a STAT3‐interacting protein and, in primary breast cancer specimens, progranulin mRNA transcripts positively correlate with STAT3 gene expression signatures and a worse clinical outcome (Yeh *et al*., 2015).

This study was undertaken to ascertain whether progranulin plays a role in sustaining STAT3 signaling in CRC.

## 2 Materials and methods

### 2.1 Patients and samples

Paired tissue samples were taken from the tumor area and the macroscopically unaffected, adjacent, colonic mucosa of 26 patients who underwent colon resection for sporadic CRC (all with TNM stages II–III) at the Tor Vergata University Hospital (Rome, Italy). No patients received radiotherapy or chemotherapy before undergoing surgery. The human studies were approved by the local ethics committee, and each patient gave written informed consent. The study methodologies conformed to the standards set by the Declaration of Helsinki.

### 2.2 Isolation of tumor‐infiltrating leukocytes from human CRC samples

Tumor‐infiltrating leukocytes were isolated from CRC samples using dithiothreitol (DTT)–ethylenediaminetetraacetic acid (EDTA) and collagenase method as previously described (De Simone *et al*., 2015). Briefly, pieces of tumor tissue were dissected from surgical specimens within 1 h of resection and washed in Hank's balanced salt solution (HBSS) containing 1 mm DTT and antibiotics for 15 min at room temperature to remove mucus. Samples were then minced and incubated in HBSS containing 1 mm EDTA and antibiotics for 45 min at 37 °C to remove epithelial cells. After two washes in HBSS, samples were incubated in type D collagenase (0.75 mg·mL^−1^, Roche Diagnostics, Monza, Italy) for 1 h at 37 °C. After collagenase digestion, media containing the mononuclear cells were collected and washed twice in HBSS. Subsequently, the pellets were resuspended in RPMI 1640 and then layered on a Percoll density gradient as previously described (Monteleone *et al*., 1997) to isolate tumor‐infiltrating leukocytes (TILs). The isolated cells were counted and checked for viability using 0.1% trypan blue (viability ranged from 90% to 98%).

### 2.3 RNA extraction, cDNA preparation, and real‐time PCR

Total RNA was extracted from human colon specimens using PureLink Purification technology (Thermo Fisher Scientific, Milan, Italy). A constant amount of RNA (1 μg/sample) was reverse‐transcribed into complementary DNA (cDNA), and 1 μL of cDNA/sample was then amplified by real‐time PCR using iQ SYBR Green Supermix (Bio‐Rad Laboratories, Milan, Italy). Primers were as follows: progranulin: FWD: 5′‐TCTGTAGTCTGAGCGCTACCC‐3′; REV: 5′‐GTTAAGGCCACCCAGCTCAC‐3′. β‐actin: FWD: 5′‐AAGATGACCCAGATCATGTTTGAGACC‐3′, REV: 5′‐AGCCAGTCCAGACGCAGGAT‐3′. RNA expression was calculated relative to the housekeeping β‐actin gene on the base of the ΔΔ*C*_t_ algorithm.

### 2.4 Progranulin silencing by progranulin antisense oligonucleotide

The custom 20mer antisense oligonucleotide (ASO) (5′‐CCACATGGTCTGCCTGCGTC‐3′) targeting both human and mouse progranulin and a scrambled (Scr) ASO were designed following previously reported guidelines (Chan *et al*., 2006; Kurreck, 2003) and synthesized by Integrated DNA Technologies (Leuven, Belgium). Progranulin ASO was used at concentrations ranging from 50 to 400 nm. Scrambled (Scr) ASO was used at concentrations equal to the highest dose of progranulin ASO in the same experiment.

### 2.5 Cell culture

All reagents were from Sigma‐Aldrich (Milan, Italy) unless specified. The human CRC cell lines HCT‐116 and HT‐29 were obtained from the American Type Culture Collection (ATCC, Manassas, VA, USA) and maintained in McCoy's 5A medium supplemented with 10% fetal bovine serum (FBS) and 1% penicillin/streptomycin (P/S) (both from Lonza) in a 37 °C, 5% CO_2_, fully humidified incubator. The human normal colonic epithelial cell line HCEC‐1CT was obtained from EVERCYTE GmbH (Vienna, Austria) and maintained in ColoUp medium (EVERCYTE GmbH) in a 37 °C, 5% CO_2_, fully humidified incubator. Cell lines were recently authenticated by STR DNA fingerprinting using the PowerPlex 18D System kit according to the manufacturer's instructions (Promega, Milan, Italy). The STR profiles of all the cell lines matched the known DNA fingerprints. Freshly isolated TILs were resuspended and cultured in complete RPMI 1640 medium and cell‐free supernatants harvested after 48 h.

To investigate whether IL‐6, IL‐22, TNF‐α, and IL‐17A modulate progranulin expression as well as STAT3, NF‐kB/p65, p38, and ERK activation, HCEC‐1CT cells were stimulated with recombinant human IL‐6 (Peprotech, London, UK), IL‐22 (R&D Systems, Minneapolis, MN, USA), TNF‐α (R&D Systems), or IL‐17A (Peprotech) (all used at 25 ng·mL^−1^) for 0.25–24 h. Progranulin expression and STAT3, NF‐kB/p65, p38, and ERK activation were assessed by western blotting. To determine the contribution of NF‐kB/p65, p38, and ERK signaling pathways on TNF‐α‐induced progranulin expression, HCEC‐1CT cells were pre‐incubated with either DMSO (vehicle) or specific inhibitors of NF‐kB/p65 (BAY 11‐7082, Santa Cruz Biotechnology, Inc., Dallas, TX, USA), p38 (SB202190, EMD Millipore, Milan, Italy), and ERK (PD98059, Calbiochem, Burlington, MA, USA) for 1 h and then stimulated with TNF‐α for further 8 h. In parallel, HT‐29 cells were treated with either DMSO or the above‐indicated inhibitors for 8 h. Progranulin expression and NF‐kB/p65, p38, and ERK activation were assessed by western blotting.

To investigate the role of progranulin in CRC cell growth, HCT‐116 and HT‐29 cells were transfected with either Scr or progranulin ASO for 24 h using Opti‐MEM medium and Lipofectamine 3000 reagent (both from Life Technologies, Milan, Italy) following the manufacturer's instructions. Cells were then washed with PBS and re‐cultured with fresh McCoy's 5A medium supplemented with 1% FBS and P/S for further 24 h. In parallel experiments, HCT‐116 and HT‐29 cells were transfected or not with either Scr or progranulin ASO and then cultured in the presence of TIL‐derived supernatants (used at 1:20 final dilution) for further 24 h.

To examine the levels of secreted progranulin in the supernatants of cell cultures, HCEC‐1CT, HCT‐116, and HT‐29 single‐cell suspensions were plated at 2 × 10^5^ cells/mL/well in 12‐well culture dishes and allowed to adhere overnight. Afterward, cells were washed with PBS and re‐cultured with fresh media. After 24 h, cell‐free supernatants were harvested and used for western blotting analysis. To address whether defects in progranulin knockdown cells were rescued by addition of progranulin, HCT‐116 cells were transfected with either Scr or progranulin ASO for 24 h and then cultured with or without 200 μg·mL^−1^ recombinant human progranulin (2420‐PG, R&D Systems, Minneapolis, MN) for further 24 h. Cell proliferation and protein expression were assessed by BrdU assay and western blotting, respectively.

To investigate the role of progranulin in CRC cell survival, HCT‐116 and HT‐29 cells were transfected with either Scr or progranulin ASO and then cultured in the presence or absence of different doses of the chemotherapeutic drugs 5‐fluorouracil (5‐FU, 6.25–100 μm), oxaliplatin (3.125–50 μm), camptothecin‐11 (CPT‐11, 0.625–10 μm), or DMSO (vehicle) for further 48–60 h. At the end, cell viability was evaluated by flow cytometry. In some experiments, HCT‐116 and HT‐29 cells were pretreated with the pan‐caspase inhibitor Q‐VD‐OPH (R&D Systems, used at 10 μm) or DMSO (vehicle) for 1 h and then transfected with Scr or progranulin ASO. Cell death was assessed by flow cytometry after 24–60 h.

### 2.6 Western blotting and Immunoprecipitation

Protein extracts were prepared and run as described elsewhere (Stolfi *et al*., 2008). Blots were incubated with antibodies against p‐STAT3 Tyr705 (sc‐8059), STAT3 (sc‐8019), CDC25A (sc‐7389), cyclin D1 (sc‐20044), cyclin D3 (sc‐182), p‐CDK2 (Thr‐14/Tyr‐15) (sc‐28435‐R), CDK2 (sc‐6248), Bcl‐2 (sc‐509), BCL‐x_L_ (sc‐8392), XIAP (sc‐55550), survivin (sc‐17779), SHP‐1 (sc‐7289), SHP‐2 (sc‐7384), c‐FLIP (sc‐5276) (1 : 500 final dilution, all from Santa Cruz Biotechnology, Inc.), progranulin (1 : 1000 final dilution, PA5‐27275, Thermo Fisher Scientific), and caspase‐3 (1:1000 final dilution, #9662, Cell signaling Technology, Danvers, MA, USA) followed by a secondary antibody conjugated to horseradish peroxidase (1 : 20 000, Dako, Santa Clara, CA, USA). After analysis, each blot was stripped and incubated with a mouse anti‐human monoclonal β‐actin antibody (1:5000 final dilution, A544) to ascertain equivalent loading of the lanes. For immunoprecipitation, 2 mg of whole‐cell lysates was incubated for 2 h at 4 °C with 2 μg of a rabbit anti‐STAT3 antibody (sc‐482) or a mouse anti‐JAK2 antibody (sc‐390539, both from Santa Cruz Biotechnology, Inc). To investigate whether progranulin affected the interaction of STAT3 with SHP‐1 and/or SHP‐2, 2 mg of whole‐cell lysates obtained from HCT‐116 cells transfected with either Scr or progranulin ASO was incubated for 2 h at 4 °C with 2 μg of a rabbit anti‐STAT3 antibody (sc‐482). The immunocomplexes were then absorbed for 2 h at 4 °C on prewashed bovine serum albumin‐blocked protein A agarose beads (EMD Millipore) and finally washed three times in wash buffer (25 mm Tris/HCl [pH 7.5], 125 mm NaCl, 1% glycerol, 1 mm MgCl_2_, 0.5% Igepal CA‐630) supplemented with complete protease inhibitor cocktail (Roche Diagnostics). Immunocomplexes were resuspended in Laemmli buffer, boiled at 75 °C for 5 min, separated by SDS‐polyacrylamide gel electrophoresis, and immunoblotted with antibodies against progranulin (PA5‐27275, Thermo Fisher Scientific), SHP‐1 (sc‐7289) or SHP‐2 (sc‐7384). The same membranes were stripped and re‐probed to detect STAT3 (sc‐8019 or sc‐482, both from Santa Cruz Biotechnology, Inc.) and β‐actin. In parallel, equal volumes of boiled immunocomplexes derived from either Scr or progranulin ASO lysates were dot‐blotted onto a nitrocellulose membrane and probed with either RPTPα (sc‐19116, Santa Cruz Biotechnology, Inc), SHP‐1 (sc‐7289), SHP‐2 (sc‐7384), or STAT3 (sc‐8019) antibodies. Computer‐assisted scanning densitometry (Image‐Lab 5.2.1, Bio‐Rad Laboratories, Milan, Italy) was used to analyze the intensity of the immunoreactive bands.

### 2.7 Assessment of cell proliferation, cycle distribution, and death

Cell growth was evaluated by using a commercially available 5‐bromodeoxyuridine (BrdU) assay kit (Roche Diagnostics). Briefly, 5000 cells were cultured in 96‐well microplates and allowed to adhere overnight. Five‐bromodeoxyuridine was added to the cell cultures 6 h before the end of the treatments and cell growth was evaluated by ELISA.

For analysis of cell cycle distribution, cells were transfected with either Scr or progranulin ASO (both used at 200 nm). After 24 h, cells were washed with PBS and re‐cultured with fresh medium containing 1% FBS and P/S for further 24 h. At the end, cells were pulsed with 10 mol·L^−1^ BrdU for 60 min, fixed in 70% cold ethanol, and stored at 20 °C for at least 3 h. DNA was denatured in 2 mol·L^−1^ HCl, and cells stained with anti‐BrdU monoclonal antibody (Roche Diagnostics) followed by a FITC‐conjugated secondary anti‐mouse antibody (Molecular Probes, Milan, Italy). After incubation with 100 g·mL^−1^ PI, cells were analyzed by flow cytometry.

To assess cell death, cells were either left untreated or transfected with either Scr or progranulin ASO as indicated in the cell culture section. Cells were then collected, washed twice in PBS, stained with FITC‐Annexin V (AV, 1:100 final dilution, Immunotools, Friesoythe, Germany) according to the manufacturer's instructions, and incubated with 5 μg·mL^−1^ propidium iodide (PI) for 30 min at 4 °C. Fluorescence was then measured using the FL‐1 and FL‐3 channels of Gallios (Beckman Coulter, Milan, Italy) flow cytometer. Viable cells were considered as AV−/PI− cells, apoptotic cells as AV+/PI− cells, while secondary necrotic cells were characterized by AV+/PI+ positive staining.

### 2.8 Organ culture

Organ culture experiments were carried out as previously described (Stolfi *et al*., 2014). Briefly, CRC explants were placed on Millicell inserts (EMD Millipore) in a 6‐well plate containing RPMI‐1640 medium supplemented with 10% FBS, 1% P/S, and 50 μg·mL^−1^ gentamycin in the presence of either Scr or progranulin ASO (both used at 200 nm) for 24 h. The culture was performed in an organ culture chamber at 37 °C in a 5% CO_2_/95% O_2_ atmosphere.

### 2.9 Immunohistochemistry

Cryosections of human CRC explants were stained with primary antibodies directed against p‐STAT3 Tyr705 (#9661, Cell Signaling Technology), Ki‐67 (sc‐101861, Santa Cruz Biotechnology, Inc.), and progranulin (PA5‐27275, Thermo Fisher Scientific). Isotype control‐stained sections were prepared under identical immunohistochemical conditions replacing the primary antibody with a rabbit normal IgG control antibody (R&D Systems, Minneapolis, MN). Positive cells were visualized using MACH4 Universal HRP‐Polymer kit with DAB (Biocare Medical, Pacheco, CA) and subsequently counted in 5 high power fields from each slide. Sections were analyzed by LEICA DMI4000 B microscope using LEICA application suite software (V4.6.2).

### 2.10 Immunofluorescence

HCT‐116 cells were grown on glass coverslips in multiwell plates, fixed with 4% paraformaldehyde for 10 min at 4 °C, and permeabilized with 0.1% Triton X‐100 for 10 min at room temperature. Cells were then washed with PBS; blocked in 1% bovine serum albumin, 0.1% Tween, and 2% glycine for 1 h at room temperature; and incubated with anti‐progranulin (1 : 100 final dilution, PA5‐27275) and anti‐p‐STAT3 Tyr705 (1 : 50 final dilution, sc‐8059) antibodies overnight at 4 °C. Subsequently, cells were rinsed with PBS and incubated with Alexa Fluor 546 (goat anti‐mouse) and Alexa Fluor 488 (donkey anti‐rabbit) (both from Life Technologies) for p‐STAT3 Tyr705 and progranulin detection, respectively, for 1 h at room temperature in the dark. After rinsing in PBS, cells were mounted using Prolong gold antifade reagent with 4′,6‐diamidino‐2‐phenylindole (Life Technologies) and visualized with a Zeiss LSM 800 Confocal Laser Scanning Microscope equipped with a 63 × objective (Carl Zeiss, Milan, Italy). Fluorescence images were processed using ZEN 2.6 (Blue Edition, Carl Zeiss).

### 2.11 Statistical analysis

Parametric data were analyzed using the two‐tailed Student's *t*‐test for comparison between two groups or one‐way analysis of variance (ANOVA) followed by Tukey's post hoc test for multiple comparisons. Nonparametric data were analyzed using the Mann–Whitney *U*‐test for comparison between two groups. Significance of correlation was determined using the Pearson's test. Significance was defined as *P*‐values < 0.05.

## 3 Results

### 3.1 Progranulin is upregulated in human CRC and correlates with STAT3 activation

**Figure 1:**
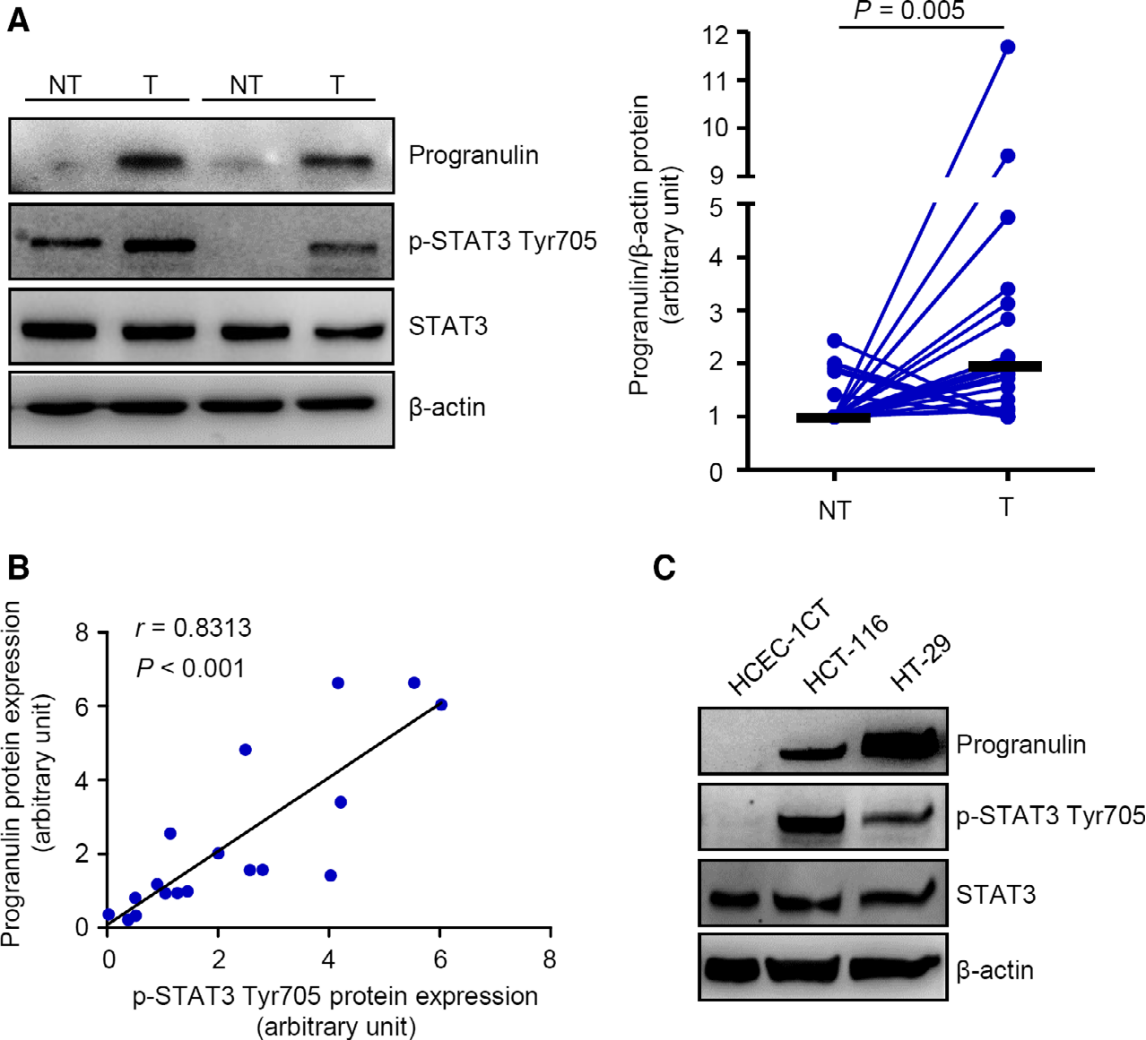
Progranulin expression is upregulated in human CRC and correlates with STAT3 activation. (A) Total proteins extracted from both tumoral (T) and nontumoral (NT) areas of two patients with sporadic CRC were evaluated for progranulin, p‐STAT3 Tyr705, and STAT3 expression by immunoblotting. β‐actin was used as loading control. The figure is representative of twenty‐six separate pairs in which similar results were obtained. Right inset. Quantitative analysis of progranulin/β‐actin protein ratio in total extracts of T and NT tissues taken from twenty‐six patients with sporadic CRC, as measured. Each point represents the value, assessed by densitometry scanning of western blots, in a single patient (*n* = 26). Horizontal bars indicate median value. Differences among groups were compared using the Mann–Whitney *U*‐test. (B) Analyses of correlation between the expression levels of progranulin and p‐STAT3 Tyr705 in the tumor areas of patients who underwent surgery for sporadic CRC (*P* < 0.001, Pearson's test coefficient r = 0.8313). (C) Total proteins extracted from two CRC cell lines (i.e., HCT‐116 and HT‐29) and from the normal colon epithelial cell line HCEC‐1CT were evaluated for progranulin, p‐STAT3 Tyr705, and STAT3 expression by immunoblotting. β‐actin was used as loading control. One of two representative experiments in which similar results were obtained is shown.

Progranulin expression and STAT3 phosphorylation on the tyrosine 705 residue were evaluated by immunoblotting in proteins extracted from matched pairs of human CRC and nontumor adjacent tissues. Progranulin protein content was significantly increased in CRC samples as compared with the nontumor mucosa, as confirmed by densitometry analysis of the blots (Fig. 1A), and positively correlated with p‐STAT3 Tyr705 expression (*r* = 0.8313, *P* < 0.001) (Fig. 1B). Analysis of progranulin mRNA in paired tissue samples taken from the tumor area and the macroscopically unaffected, adjacent, colonic mucosa of the same patients whose specimens were considered for protein analysis, showed more progranulin transcripts in the tumors compared to nontumor areas (Fig. S1). Consistent with the above data, elevated levels of progranulin, along with a strong expression of p‐STAT3 Tyr705, were observed in the human CRC cell lines HCT‐116 and HT‐29, whereas both proteins were barely detectable in the human normal colonic epithelial cell line HCEC‐1CT (Fig. 1C). As progranulin is a growth factor‐like protein and can be secreted (Arechavaleta‐Velasco *et al*., 2017), we also evaluated progranulin expression in the culture supernatants of HCEC‐1CT, HCT‐116, and HT‐29 cells. Levels of the secreted protein reflected those detected in the cell lysates (Fig. S2).

### 3.2 Progranulin interacts with and positively regulates STAT3 activation in CRC cells

**Figure 2:**
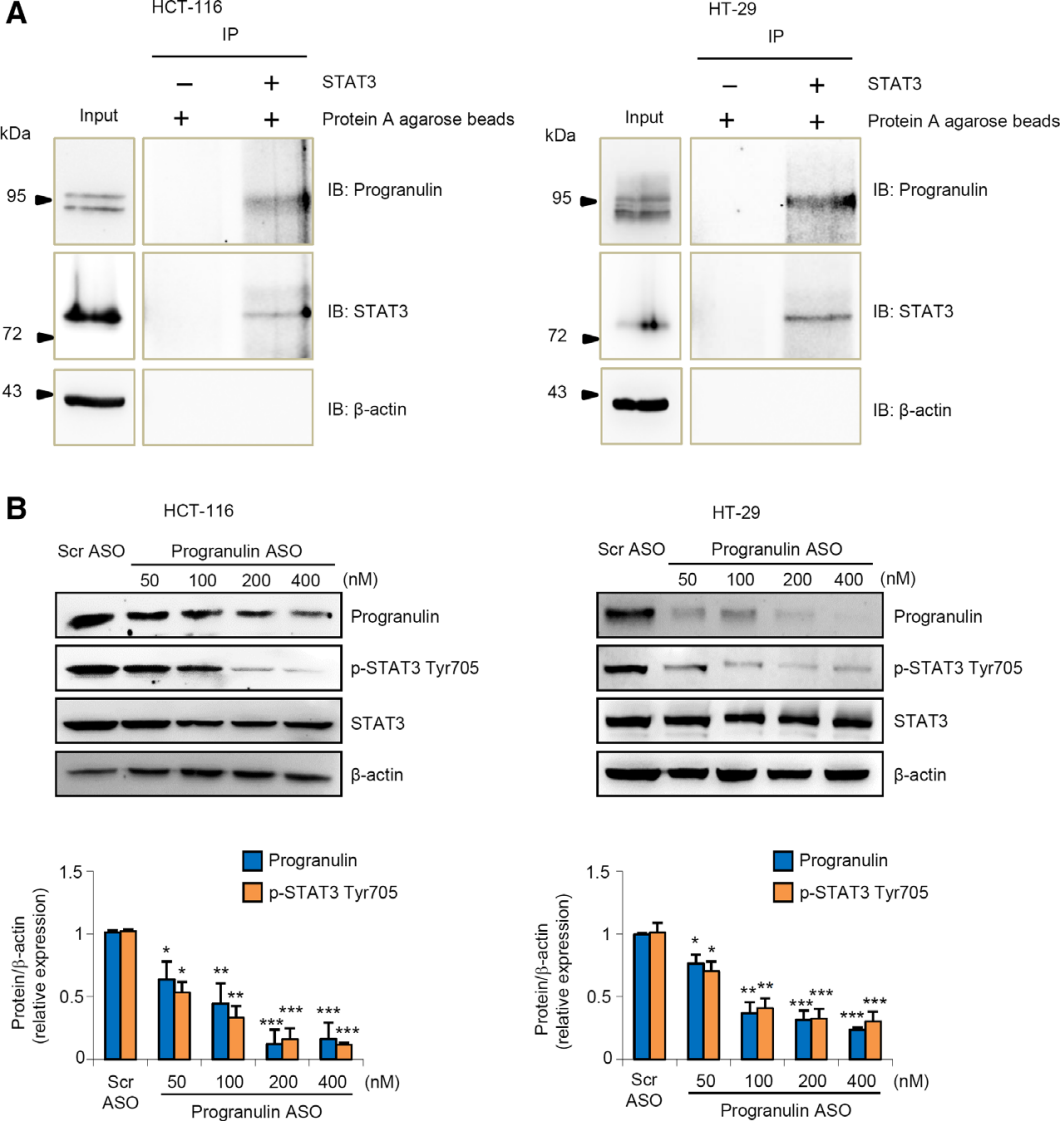
Progranulin interacts with and positively regulates STAT3 activation in CRC cells. (A) Total proteins extracted from HCT‐116 and HT‐29 cells were immunoprecipitated by an anti‐human STAT3 antibody and then subjected to immunoblotting analysis using progranulin, STAT3, and β‐actin antibodies. One of three representative experiments in which similar results were obtained is shown. (B) Progranulin ASO downregulates progranulin and p‐STAT3 Tyr705 protein expression in CRC cells. HCT‐116 and HT‐29 cells were transfected with either scrambled (Scr) antisense oligonucleotide (ASO) (used at 400 nm) or increasing doses of progranulin ASO as indicated. Progranulin, p‐STAT3 Tyr705, and β‐actin were analyzed by immunoblotting. One of three representative experiments is shown together with the densitometry analysis in the lower insets. Values are expressed in arbitrary units and are the mean ± SEM. Differences were compared using the two‐tailed Student's *t*‐test (Scr ASO‐transfected cells vs progranulin ASO‐transfected cells, *P < 0.05, **P < 0.01, ***P < 0.001).

**Figure 3:**
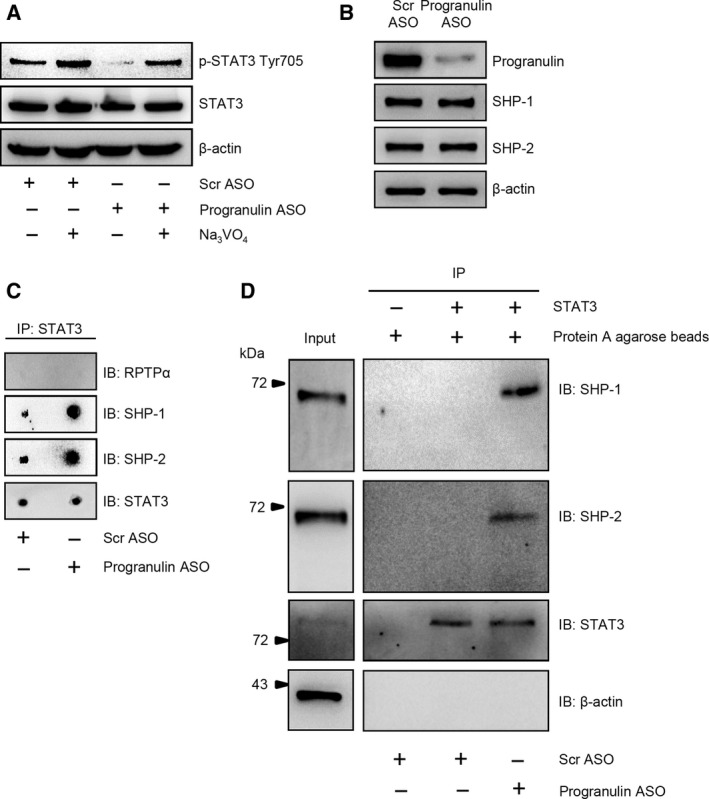
Effects of progranulin on protein tyrosine phosphatase (PTP)‐mediated STAT3 dephosphorylation. (A) The addition of the general PTP inhibitor Na_3_ VO_4_ to HCT‐116 cells cultures reduces the progranulin silencing‐mediated STAT3 Tyr705 dephosphorylation. HCT‐116 cells were transfected with either scrambled (Scr) or progranulin antisense oligonucleotide (ASO) (both used at 200 nm). After 24 h, cells were washed with PBS and cultured with or without Na_3_ VO_4_ for 2 h. At the end, total extracts were prepared and the expression of p‐STAT3 Tyr705 and STAT3 assessed by western blotting. β‐actin was used as loading control. One of three representative experiments where similar results were obtained is shown. (B) Progranulin knockdown does not affect the expression of the STAT3 targeting PTPs SHP‐1 and SHP‐2. Total proteins extracted from either Scr ASO‐ or progranulin ASO‐transfected HCT‐116 cells were evaluated for progranulin, SHP‐1, and SHP‐2 expression by western blotting. β‐actin was used as loading control. One of three representative experiments where similar results were obtained is shown. (C,D) Progranulin impairs the interaction of SHP‐1 and SHP‐2 with STAT3. Total proteins extracted from either Scr ASO‐ or progranulin ASO‐transfected HCT‐116 cells were immunoprecipitated by an anti‐human STAT3 antibody. An aliquot of proteins was dot‐blotted on nitrocellulose and incubated with either the indicated PTP or STAT3 antibodies (C). The remaining immunoprecipitates were separated by SDS/PAGE and then subjected to immunoblotting analysis using either SHP‐1 or SHP‐2 antibodies. At the end, blots were stripped and incubated with either a second anti‐human STAT3 or β‐actin antibodies. One of three representative experiments in which similar results were obtained is shown (D).

The observation that progranulin and p‐STAT3 Tyr705 expression strongly correlate in human CRC prompted us to investigate whether progranulin might affect STAT3 oncogenic activity. Initially, we performed immunoprecipitation of whole‐cell extracts from HCT‐116 and HT‐29 cells using an anti‐STAT3 antibody followed by immunoblotting analysis with an anti‐progranulin antibody and showed that progranulin physically interacted with STAT3 in CRC cells (Fig. 2A). Next, we tested whether progranulin silencing could reduce STAT3 phosphorylation. Treatment of HCT‐116 and HT‐29 cells with progranulin ASO significantly reduced progranulin expression and STAT3 Tyr705 phosphorylation in a dose‐dependent manner (Fig. 2B). Immunofluorescence experiments revealed that progranulin co‐localized with p‐STAT3 Tyr705 mainly in the cytoplasm (Fig. S3). To figure out how progranulin positively affected STAT3 Tyr705 phosphorylation, we first investigated whether progranulin associated with JAK2, a key STAT3 Tyr705 targeting kinase (Rawlings *et al*., 2004), someway promoting its activity. However, we were not able to see any interaction between progranulin and JAK2 in HCT‐116 cells (Fig. S4). Further work indicated that progranulin knockdown‐mediated inhibitory effect on STAT3 Tyr705 phosphorylation was reverted by the addition of the protein tyrosine phosphatase inhibitor Na_3_VO_4_ (Fig. 3A), thus suggesting a role for progranulin in modulating STAT3 dephosphorylation. More in‐depth experimentation revealed that intracellular progranulin restrained the ability of key STAT3 tyrosine phosphatases (i.e., SHP‐1 and SHP‐2) (Kim *et al*., 2018) to interact with the transcription factor (Fig. 3B–D), likely contributing to STAT3 hyper‐activation.

### 3.3 NF‐kB/p65 signaling affects progranulin expression in human normal and cancerous colonic cell lines

**Figure 4:**
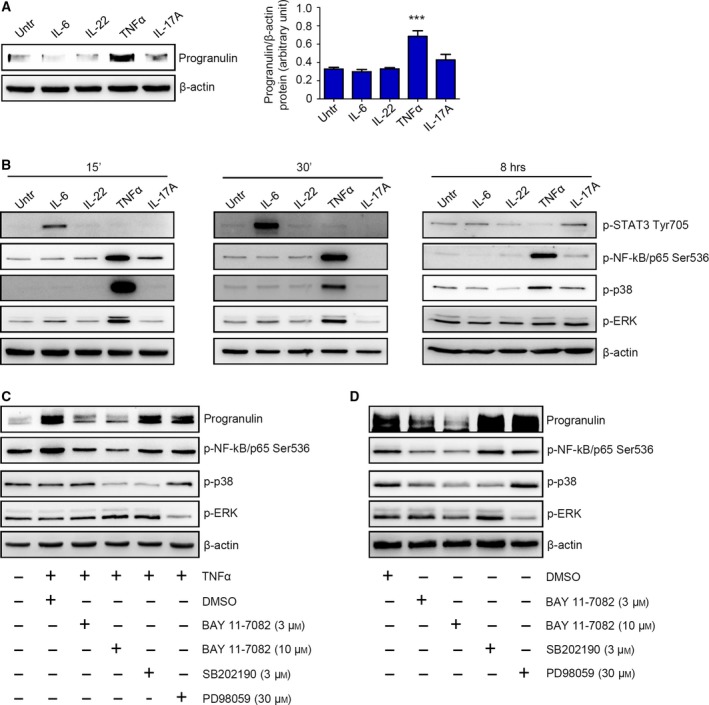
NF‐kB/p65‐mediated regulation of progranulin expression in human normal and cancerous colonic cell lines. (A) Representative western blotting showing progranulin expression in HCEC‐1CT cells stimulated or not with IL‐6, IL‐22, TNF‐α, and IL‐17A (all used at 25 ng·mL^−1^) for 8 h. β‐actin was used as a loading control. One of four representative experiments in which similar results were obtained is shown. Right inset. Quantitative analysis of progranulin/β‐actin protein ratio in total extracts of HCEC‐1CT cells stimulated or not with IL‐6, IL‐22, TNF‐α, and IL‐17A for 8 h, as measured by densitometry scanning of western blots. Values are expressed in arbitrary units and are the mean ± SEM of four experiments. Differences among groups were compared using one‐way analysis of variance (ANOVA) followed by Tukey's post hoc test. TNF‐α‐stimulated cells vs untreated cells, ***P < 0.001. (B) Representative western blotting showing p‐STAT3 Tyr705, p‐NF‐kB/p65 Ser536, p‐p38, and p‐ERK expression in HCEC‐1CT cells stimulated or not with IL‐6, IL‐22, TNF‐α, and IL‐17A (all used at 25 ng·mL^−1^) for 15 min (left panels), 30 min (middle panels), and 8 h (right panels). β‐actin was used as a loading control. One of three representative experiments in which similar results were obtained is shown. (C) Representative western blotting showing progranulin, p‐NF‐kB/p65 Ser536, p‐p38, and p‐ERK expression in HCEC‐1CT cells either left untreated or stimulated with TNF‐α (used at 25 ng·mL^−1^) for 8 h in the presence of NF‐kB/p65 inhibitor (BAY 11‐7082), p38 inhibitor (SB202190), ERK inhibitor (PD98059), or DMSO (vehicle) as indicated. β‐actin was used as a loading control. One of three representative experiments in which similar results were obtained is shown. (D). Representative western blotting showing progranulin, p‐NF‐kB/p65 Ser536, p‐p38, and p‐ERK expression in HT‐29 cells cultured for 8 h in the presence of NF‐kB/p65 inhibitor (BAY 11‐7082), p38 inhibitor (SB202190), ERK inhibitor (PD98059), or DMSO (vehicle) as indicated. β‐actin was used as a loading control. One of three representative experiments in which similar results were obtained is shown.

In order to elucidate the factors/mechanisms involved in the modulation of progranulin expression, HCEC‐1CT cells were cultured in the presence of cytokines (i.e., IL‐6, IL‐22, TNF‐α, and IL‐17A), which are over‐produced within the neoplastic areas of CRC patients (De Simone *et al*., 2015). TNF‐α, but not the other cytokines, significantly enhanced progranulin expression at 8 and 24 h (Fig. 4A and Fig. S5). Analysis of the signaling pathways activated by such cytokines revealed that 15‐min treatment of HCEC‐1CT cells with TNF‐α resulted in a strong activation of NF‐kB/p65, p38, and ERK, whereas IL‐17A slightly increased NF‐kB/p65 activation (Fig. 4B). Further evaluation at 0.5, 1, and 8 h of cytokine stimulation showed that cells stimulated with TNF‐α, but not with IL‐17A, maintained NF‐kB/p65 activation (Fig. 4B and not shown). One‐hour treatment of HCEC‐1CT cells with IL‐17A slightly increased p38 phosphorylation (not shown). In contrast, we were not able to detect any p‐ERK upregulation in cells stimulated with IL‐17A at all the time points considered (Fig. 4B and not shown).

Pretreatment of HCEC‐1CT cells with BAY 11‐7082, a chemical inhibitor of NF‐kB/p65, almost completely abolished the TNF‐α‐induced upregulation of progranulin (Fig. 4C). In contrast, no effect on progranulin expression was seen in cells cultured with either p38 (i.e., SB202190) or ERK (i.e., PD98059) inhibitors (Fig. 3C). In line with such observation, inhibition of NF‐kB/p65 activation, but neither p38 nor ERK, reduced progranulin levels in unstimulated HT‐29 cells (Fig. 4D). Collectively, the above results indicate that NF‐kB/p65 activation sustains progranulin expression in human CRC cells.

### 3.4 Progranulin knockdown hampers STAT3 oncogenic functions in CRC cells

**Figure 5:**
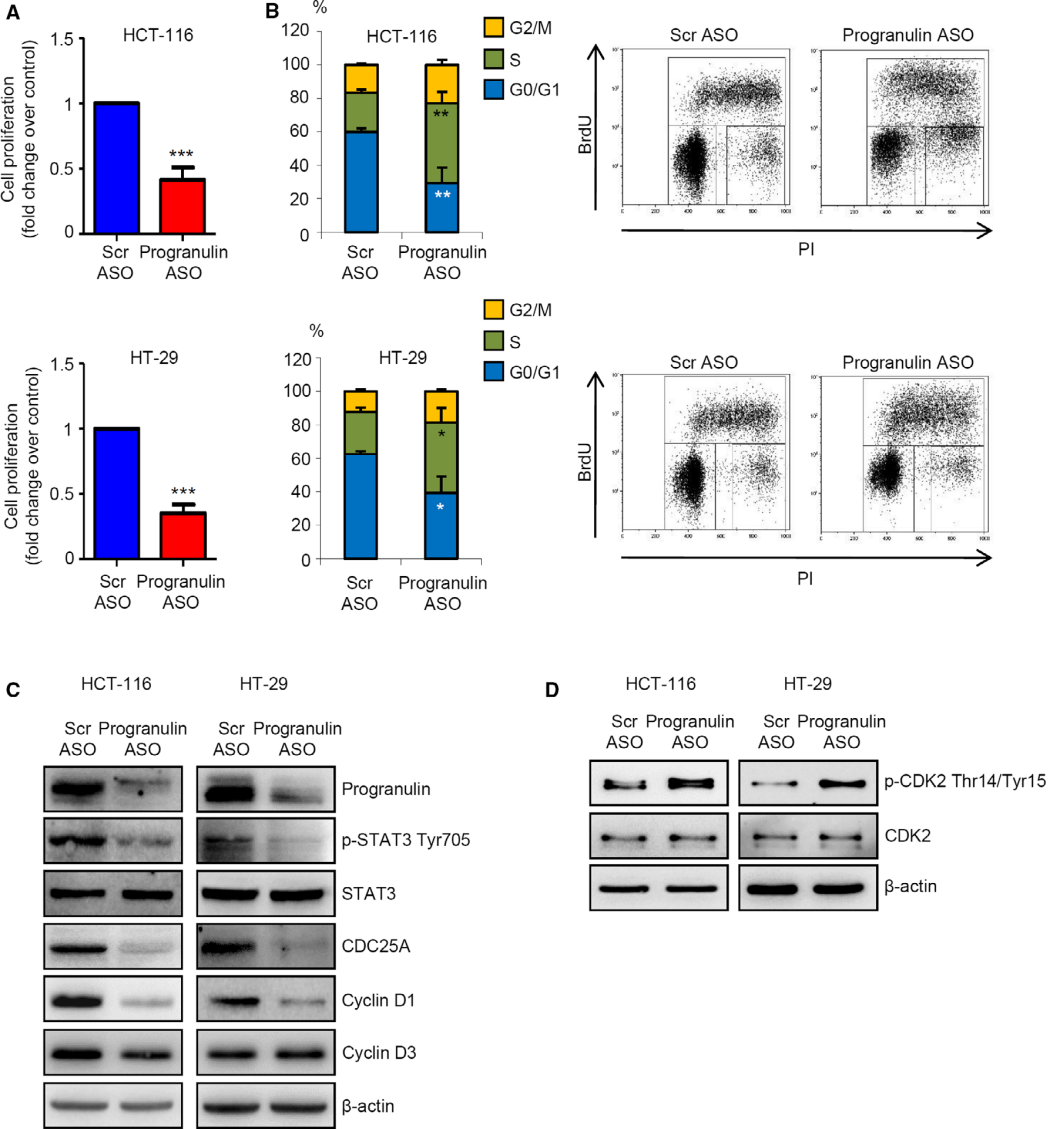
Progranulin knockdown negatively affects STAT3 oncogenic activity and CRC cell growth (A) Progranulin silencing impaired CRC cell proliferation. HCT‐116 and HT‐29 cells were transfected with either scrambled (Scr) or progranulin antisense oligonucleotide (ASO) (both used at 200 nm). After 24 h, cells were washed with PBS and cultured for further 24 h. Cell proliferation was assessed by 5‐bromodeoxyuridine (BrdU) proliferation assay kit. Data indicate mean ± SEM of four experiments. Differences were compared using the two‐tailed Student's *t*‐test. ***P < 0.001. (B) Progranulin ASO induces CRC cells to arrest in S phase of the cell cycle. HCT‐116 and HT‐29 cells were transfected with either scrambled (Scr) or progranulin antisense oligonucleotide (ASO) (both used at 200 nm). After 24 h, cells were washed with PBS and cultured for further 24 h. Cell cycle distribution was assessed by flow cytometry. Values are the percentages of cells in the different phases of cell cycle and indicate mean ± SD of four experiments. A significant increase in the number of cells that accumulate in S phase and a significant decrease in the number of cells in G0/G1 was seen in progranulin ASO‐transfected cells as compared with Scr ASO‐transfected cells (*P < 0.01, **P < 0.001). Differences were compared using the two‐tailed Student's *t*‐test. Right insets show representative dot‐plots of the cell cycle distribution. (C) Total proteins from HCT‐116 and HT‐29 cells treated as indicated in A were extracted and evaluated for progranulin, p‐STAT3 Tyr705, STAT3, CDC25A, cyclin D1, and cyclin D3 expression by western blotting. β‐actin was used as loading control. One of three representative experiments where similar results were obtained is shown. (D) Progranulin ASO enhances the expression of p‐CDK2. HCT‐116 and HT‐29 cells were treated as indicated in A. Phospho‐CDK2, CDK2, and β‐actin expression was analyzed by immunoblotting. One of three representative experiments in which similar results were obtained is shown.

As STAT3 is a positive regulator of CRC cell proliferation and survival, we investigated whether progranulin silencing inhibited HCT‐116 and HT‐29 cell growth. Cells transfected with progranulin ASO showed a significant reduction of 5‐bromodeoxyuridine (BrdU) incorporation as compared with cells transfected with a control ASO (Fig. 5A). Analysis of cell cycle distribution indicated that progranulin ASO‐induced CRC cell growth inhibition associated with accumulation of cells in S phase and decreased frequency of cells in G0/G1 phase of the cell cycle (Fig. 5B).

In tumors, the dual‐specificity phosphatase CDC25A and cyclin D1 (Carpenter and Lo, 2014) regulate G1/S transition and progression through the S phase (Otto and Sicinski, 2017). Transfection of HCT‐116 and HT‐29 cells with progranulin ASO markedly reduced CDC25A and cyclin D1 expression (Fig. 5C). Of note, the expression of cyclin D3, a cell cycle regulator not subjected to STAT3 control, was not affected by progranulin silencing in both cell lines (Fig. 5C). CDC25A exerts its pro‐mitogenic functions mainly by maintaining cyclin‐dependent kinases (CDK)–cyclin complexes in a persistently activated state through the removal of inhibitory phosphate groups from tyrosine and threonine residues (Busino *et al*., 2004). Consistent with this, the progranulin ASO‐driven CDC25A downregulation resulted in a marked phosphorylation of the S phase‐associated protein CDK2 on Thr‐14 and Tyr‐15 residues (Fig. 5D). At the time point used to evaluate cell growth and cell cycle distribution (i.e., 36 h from transfection), there was no change in the viability of progranulin ASO‐treated cells (not shown), indicating that the progranulin ASO‐mediated cell growth arrest was not secondary to cell death.

We next investigated whether defects in progranulin knockdown cells could be reverted by exogenous progranulin. Addition of progranulin did not rescue the negative effects of progranulin silencing on cell proliferation (Fig. S6A) as well as on p‐STAT3 Tyr705 and cyclin D1 expression (Fig. S6B).

**Figure 6:**
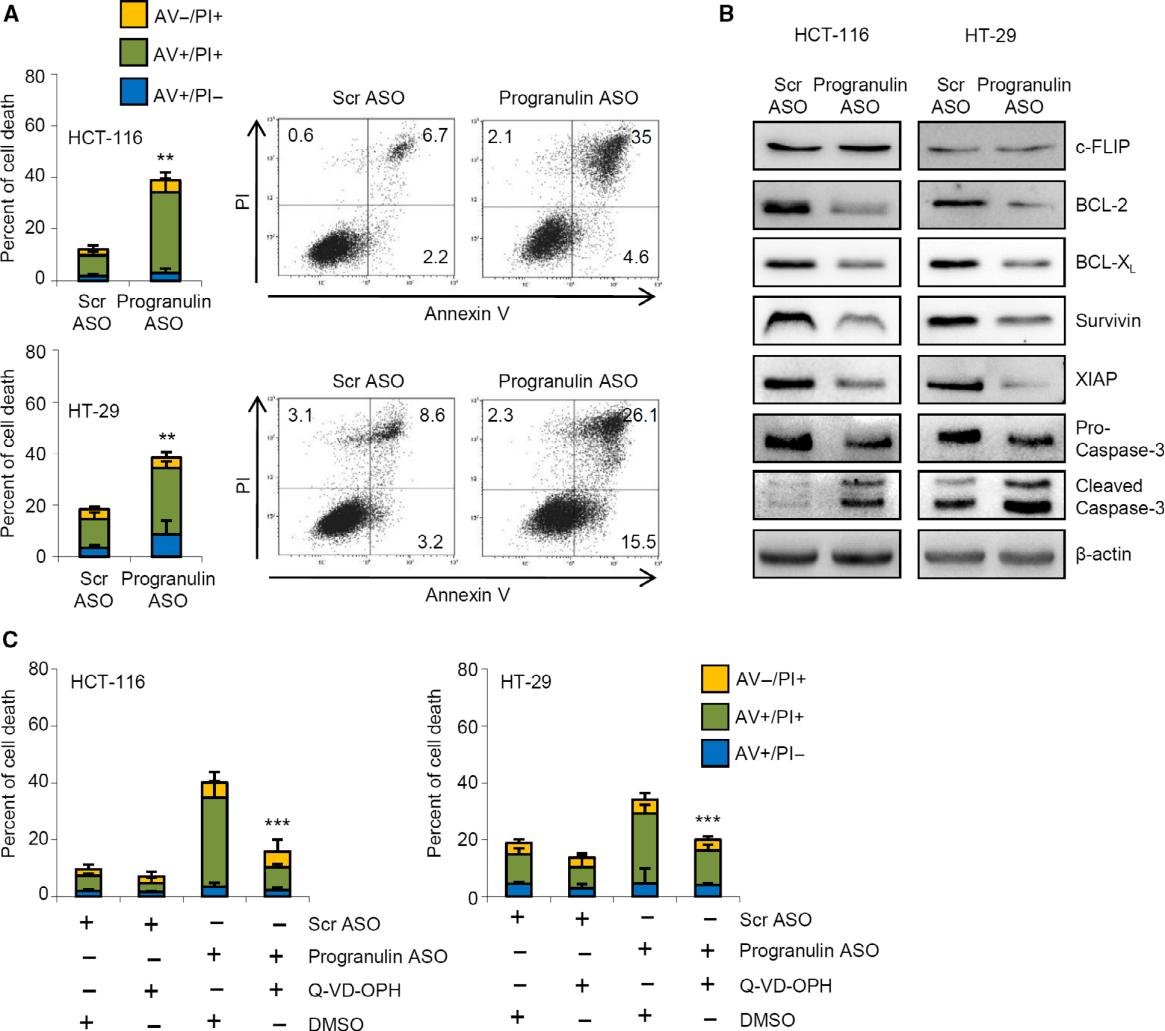
Progranulin silencing negatively affects the expression of STAT3‐related anti‐apoptotic proteins and induces CRC cell death through a caspase‐dependent mechanism. (A) Inhibition of progranulin induces CRC cell death. HCT‐116 and HT‐29 cells were transfected with either scrambled (Scr) or progranulin antisense oligonucleotide (ASO) (both used at 200 nm). After 24 h, cells were washed with PBS and cultured for further 48 and 60 h, respectively. Data indicate the percentage of cell death as assessed by flow cytometry analysis of Annexin V (AV) and/or propidium iodide (PI)‐positive cells and are expressed as mean ± SD of three experiments. Differences were compared using the two‐tailed Student's *t*‐test. **P < 0.01. Right panels. Representative dot‐plots showing the percentages of AV‐ and/or PI‐positive cells. (B) Progranulin knockdown reduced the expression of STAT3‐related anti‐apoptotic proteins. Representative western blotting of whole‐cell extracts from HCT‐116 and HT‐29 cells treated as indicated in A and evaluated for c‐FLIP, Bcl‐2, BCL‐X_L_, survivin, XIAP, pro‐caspase‐3, and cleaved caspase‐3 expression by western blotting. One of three representative experiments in which similar results were obtained is shown. (C) Pre‐incubation of HCT‐116 and HT‐29 cells with Q‐VD‐OPH abolishes progranulin ASO‐induced cell death. HCT‐116 and HT‐29 cells were pretreated with the pan‐caspase inhibitor Q‐VD‐OPH, transfected with either scrambled (Scr) or progranulin antisense oligonucleotide (ASO) (both used at 200 nm), and cultured for further 48 h (HCT‐116) and 60 h (HT‐29). Data indicate the percentage of cell death as assessed by flow cytometry analysis of Annexin V (AV) and/or propidium iodide (PI)‐positive cells and are expressed as mean ± SD of three experiments. Differences among groups were compared using one‐way analysis of variance (ANOVA) followed by Tukey's post hoc test (progranulin ASO‐transfected cells + DMSO vs progranulin ASO‐transfected cells + Q‐VD‐OPH, ***P < 0.001).

As a persistent cell cycle arrest in S phase is usually followed by the induction of programmed cell death, we next analyzed the fraction of AV/PI‐positive cells at later time points. The cell cycle perturbation of progranulin‐deficient HCT‐116 and HT‐29 cells was accompanied by increased cell death at 60 and 72 h, respectively (Fig. 6A). At the same time points, progranulin knockdown resulted in a pronounced decrease in pivotal STAT3‐related anti‐apoptotic proteins (i.e., Bcl‐2, BCL‐X_L_, survivin, and XIAP) (Fig. 6B). In contrast, no relevant change in the expression of c‐Flip, a master anti‐apoptotic regulator not subjected to STAT3 control, was seen upon treatment of cells with progranulin ASO (Fig. 6B). In line with the above results, a marked increase in cleaved caspase 3, one of the key effector molecules in the apoptotic pathway (Elmore, 2007), was detected in both CRC cell lines following progranulin inhibition (Fig. 6B). Pre‐incubation of CRC cells with the pan‐caspase inhibitor Q‐VD‐OPH almost completely reverted the progranulin ASO‐mediated cell death (Fig. 6C).

Altogether, the above findings indicate that progranulin is a positive regulator of CRC cell growth and survival.

### 3.5 Progranulin knockdown sensitizes CRC cells to chemotherapeutic drugs

**Figure 7:**
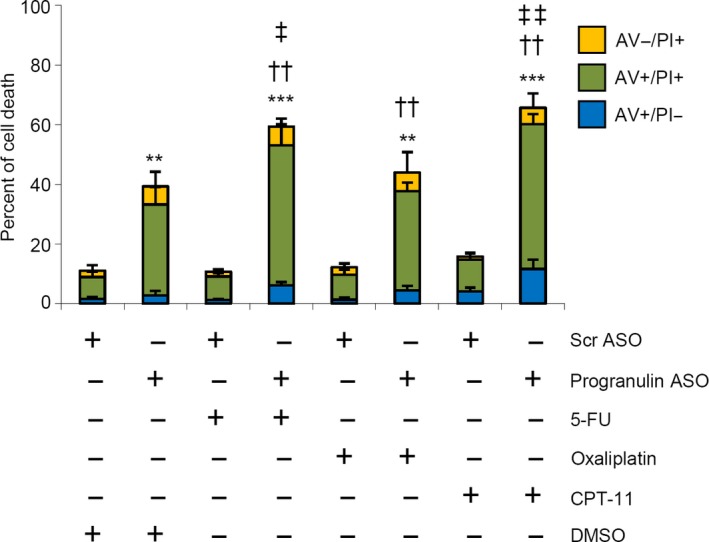
Progranulin knockdown enhances the toxicity of chemotherapeutic drugs. HCT‐116 cells were transfected with either scrambled (Scr) or progranulin antisense oligonucleotide (ASO) (both used at 200 nm). After 24 h, cells were washed with PBS and cultured with 5‐fluorouracil (5‐FU, 25 μm), oxaliplatin (12.5 μm), camptothecin‐11 (CPT‐11, 2.5 μm), or DMSO (vehicle) for further 48 h. Data indicate the percentage of cell death as assessed by flow cytometry analysis of Annexin V (AV) and/or propidium iodide (PI)‐positive cells and are expressed as mean ± SD of three experiments. Differences among groups were compared using one‐way analysis of variance (ANOVA) followed by Tukey's post hoc test (progranulin ASO‐transfected cells ± chemotherapeutics vs Scr ASO‐transfected cells + DMSO, **P < 0.01, ***P < 0.001; progranulin ASO‐transfected cells + chemotherapeutics vs chemotherapeutics alone, ††*P* < 0.001, progranulin ASO‐transfected cells + chemotherapeutics vs progranulin ASO‐transfected cells ‡*P* < 0.05, ‡‡*P* < 0.01).

Bcl‐2, Bcl‐X_L_, survivin, and XIAP have been involved in the resistance of cancer cells to chemotherapy (Ji *et al*., 2013; Maji *et al*., 2018; Miyamoto *et al*., 2014; Real *et al*., 2002). As data in Fig. 5B indicate that progranulin positively regulates such proteins, we hypothesized that knockdown of progranulin could enhance the susceptibility of CRC cells to chemotherapeutic drugs. Preliminary dose–response experiments with agents commonly used in chemotherapy for CRC [i.e., 5‐fluorouracil (5‐FU), oxaliplatin, and CPT‐11] were carried out in CRC cells to establish the appropriate concentrations of the drugs (Fig. S7). Treatment of HCT‐116 cells with each of the 3 chemotherapeutic drugs did not alter the rate of cell death (Fig. 7). As expected, progranulin ASO significantly enhanced the fraction of cell death as compared to control cells, and this effect was further increased by treatment of cells with 5‐FU and CPT‐11 (Fig. 7).

### 3.6 Progranulin silencing abrogates TIL‐derived culture supernatant‐mediated cell proliferation in CRC cells

**Figure 8:**
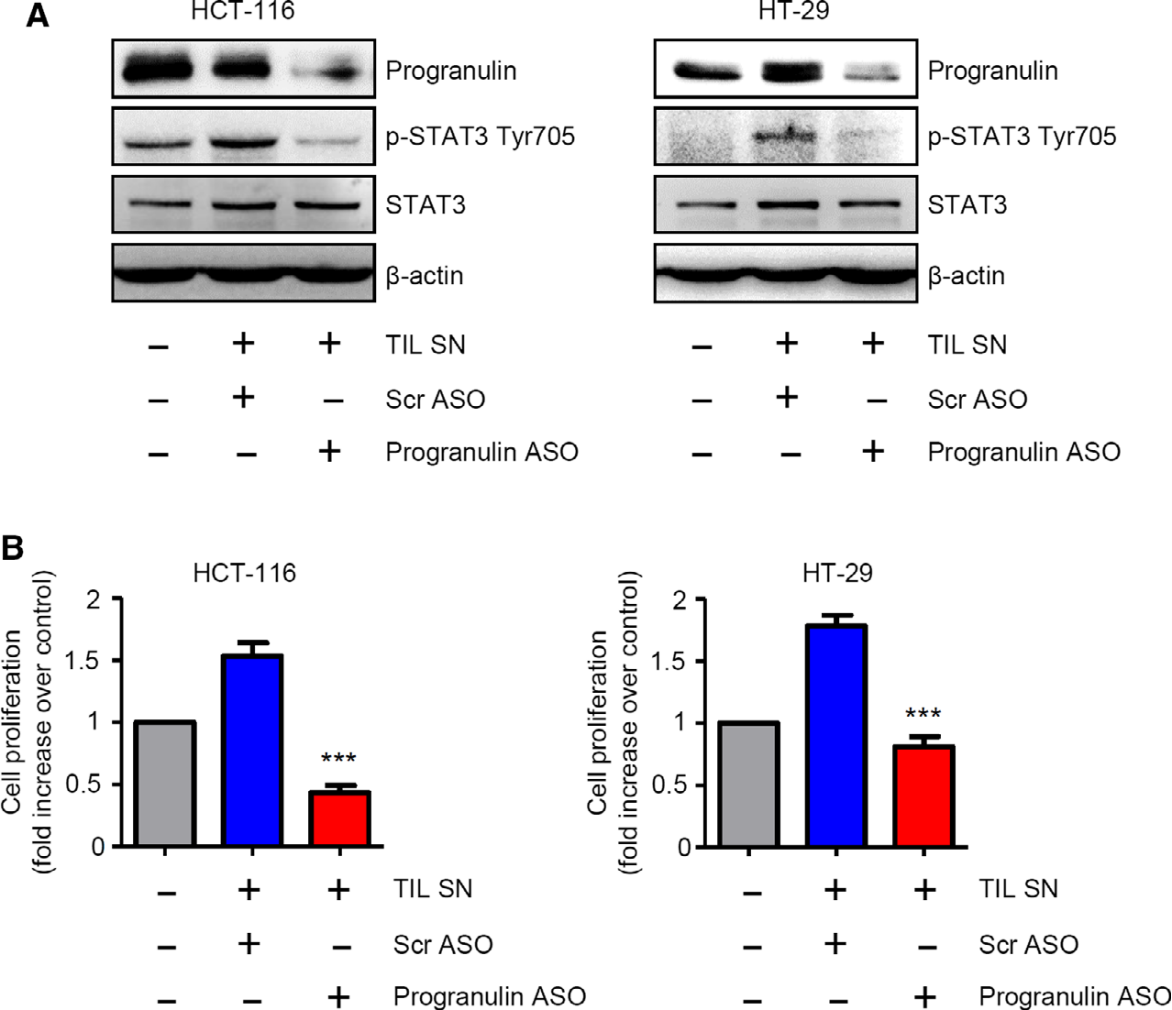
Effect of progranulin inhibition on tumor‐infiltrating leukocyte‐derived supernatant (TIL SN)‐mediated STAT3 activation and increase of CRC cell growth. (A) Progranulin silencing completely abrogates TIL SN‐driven STAT3 activation. Representative western blotting showing progranulin, p‐STAT3 Tyr705 and STAT3 expression in HCT‐116 and HT‐29 cells either left untreated or transfected with either scrambled (Scr) or progranulin antisense oligonucleotide (ASO) (both used at 200 nm) in the presence of TIL SN. β‐actin was used as loading control. One of three representative experiments in which similar results were obtained is shown. (B) Progranulin silencing completely suppresses TIL SN‐mediated increase of CRC cell proliferation. Representative histograms showing cell proliferation of HCT‐116 and HT‐29 cells treated as indicated in A. Data indicate mean ± SEM of four experiments. Differences among groups were compared using one‐way analysis of variance (ANOVA) followed by Tukey's post hoc test. Scr ASO‐transfected cells + TIL SN vs progranulin ASO‐transfected cells + TIL SN, ***P < 0.001.

Colorectal tumors often show a diffuse infiltrate of cytokine‐producing immune/inflammatory cells that contribute to disease progression in part via the activation of STAT3 signaling in transformed epithelial cells (De Simone *et al*., 2015). To link our *in vitro* observations to primary human cells, we isolated tumor‐infiltrating leukocytes (TILs) from the tumor area of patients who underwent surgery for CRC and assessed whether TIL‐derived culture supernatants could modulate STAT3 activation and cell proliferation in HCT‐116 and HT‐29 cells transfected with either progranulin or control ASO. TIL‐derived supernatants robustly increased p‐STAT3 Tyr705 expression and cell proliferation in both HCT‐116 and HT‐29 cells as compared with untreated conditions (Fig. 8A,B). Notably, such effects were totally abrogated in cells transfected with progranulin ASO, but not with Scr ASO (Fig. 8A,B).

### 3.7 Inhibition of progranulin reduces the proliferation of neoplastic cells in human CRC explants

**Figure 9:**
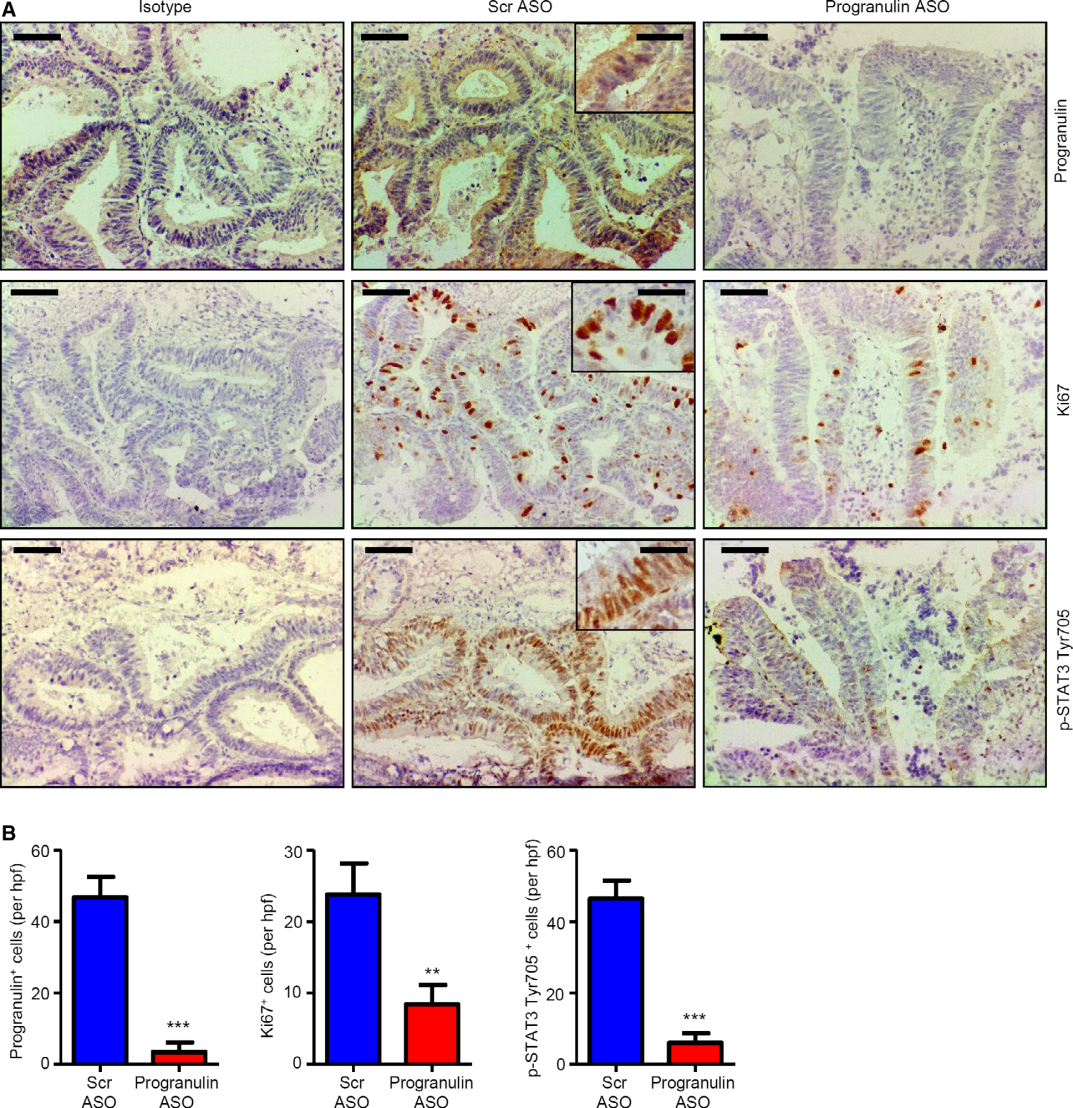
Inhibition of progranulin with the specific progranulin antisense oligonucleotide (ASO) reduces STAT3 activation and the proliferation of neoplastic cells in human CRC explants. (A) Representative pictures of progranulin‐, Ki67‐, and p‐STAT3 Tyr705‐stained sections of freshly obtained CRC explants treated with either scrambled (Scr) or progranulin antisense oligonucleotide (ASO) (both used at 400 nm) for 24 h. Isotype control stainings are also indicated. The scale bars are 40 μm. The scale bars in the insets are 10 μm. One of four representative experiments in which similar results were obtained is shown. (B) Quantification of progranulin‐, Ki67‐, and p‐STAT3 Tyr705‐positive cells in sections of freshly obtained CRC explants treated as indicated in A. Data are presented as mean values of positive cells per high power field (hpf) ± SEM of four independent experiments. Differences were compared using the two‐tailed Student's *t*‐test (Scr ASO‐ vs progranulin ASO‐treated CRC explants, **P < 0.01, ***P < 0.001).

To translate our findings *in vivo*, progranulin ASO was added to organ cultures of human CRC explants, and cell growth and STAT3 activation were analyzed after 24 h by immunohistochemistry. Consistently with results obtained in CRC cells, progranulin inhibition reduced the fraction of transformed cells expressing Ki67, a cellular marker of proliferation, as well as the number of p‐STAT3 Tyr705‐expressing cells (Fig. 9A,B).

## 4 Discussion

This study was undertaken to investigate whether progranulin sustains STAT3 hyper‐activation in CRC and whether its inhibition may represent a feasible approach to restrain STAT3 oncogenic function in such neoplasia.

Signal Transducer and Activator of Transcription 3 is the hub of multiple oncogenic pathways, and both experimental and preclinical evidence pinpoints the inhibition of STAT3 signaling as an appealing anticancer strategy (Johnson *et al*., 2018). To date, the direct targeting of STAT3 through small molecules remains the best option to achieve this goal. Unfortunately, issues related to the suboptimal efficacy and unfavorable pharmacokinetic properties of such compounds have limited their use in the clinic (Beebe *et al*., 2018). Antisense technology has recently emerged as a compelling therapeutic strategy to target difficult to hit proteins by downregulating their mRNA in several disease settings (Le *et al*., 2018; Marafini and Monteleone, 2018; Rinaldi and Wood, 2018). In particular, antitumor activity of an ASO inhibitor of STAT3 (i.e., AZD9150) was observed in two phase 1 studies in patients with highly treatment‐refractory lymphomas and non‐small cell lung cancer (Hong *et al*., 2015; Reilley *et al*., 2018). However, both approaches do not overcome the issue of potential side effects related to the broad inhibition of STAT3 function.

Here we show that progranulin expression is increased in human CRC samples relative to surrounding nontumor tissues and positively correlates with STAT3 activation. Consistent with such data, higher progranulin levels, as well as STAT3 hyper‐activation, is detectable in CRC cell lines compared with normal colonic epithelial cells. We also show that progranulin physically interacts with STAT3 in CRC cells and knockdown of progranulin with a specific ASO negatively affects the expression of p‐STAT3 Tyr705, leaving unchanged total STAT3 protein expression. Such observation suggests that progranulin might someway take part in the modulation of STAT3 phosphorylation/activation. In the effort to figure out how progranulin affects STAT3 Tyr705 phosphorylation, we here show that intracellular progranulin restrains the ability of pivotal STAT3 tyrosine phosphatases (i.e., SHP‐1 and SHP‐2) to interact with the transcription factor, thus sustaining STAT3 hyper‐activation. Of note, accumulating evidence indicates a tumor‐suppressive role for SHP‐1 and SHP‐2 and supports the use pharmacological strategies aimed at enhancing the activity of such tyrosine phosphatases in cancer cells (Fan *et al*., 2015; Huang *et al*., 2017; Qi *et al*., 2017). Thus, targeting progranulin might represent a different approach to achieve this goal. However, we cannot exclude the possibility that progranulin might affect other STAT3‐targeting tyrosine phosphatases (Kim *et al*., 2018), and such an effect has some contribution to progranulin‐mediated STAT3 hyper‐activation. Further and more in‐depth studies beyond those presented here will be needed to answer this question.

To begin to uncover the mechanism/s underlying progranulin upregulation in CRC cells, we stimulated normal colonic epithelial cells with various cytokines, which are upregulated in CRC (De Simone *et al*., 2015). Our data indicate that TNF‐α increases progranulin levels in normal colon epithelial cells via NF‐kB/p65 activation and that pharmacological inhibition of NF‐kB in CRC cell lines reduces progranulin expression. Surprisingly, we were not able to detect p‐STAT3 Tyr705 upregulation in HCEC‐1CT cells upon treatment with IL‐22, a potent STAT3 activator in colon epithelial cells (De Simone *et al*., 2015), at all the time points considered. Similarly, in apparent contrast with the literature indicating IL‐17A as a p‐ERK inducer (Fabre *et al*., 2016), no ERK activation was seen in HCEC‐1CT cells cultured with IL‐17A. However, as IL‐22 strongly activated STAT3 in HCT‐116 cells (not shown) and IL‐17A was biologically active in our system, a possible reason for such discrepancies may rely in HCEC‐1CT cell signaling mutations/defects.

Altogether, these findings suggest that NF‐kB/p65‐activating factors secreted within the tumor microenvironment can enhance progranulin synthesis and consequently promote STAT3 activation, thus adding another piece of evidence supporting the dangerous liaison between STAT3 and NF‐kB in colon carcinogenesis (Grivennikov and Karin, 2010).

Progranulin silencing mirrors the effect of STAT3 inhibition on CRC cell growth. Indeed, knockdown of progranulin in CRC cell lines associates with reduced proliferation and arrest of cells in the S phase of the cell cycle, as well as with a marked and specific downregulation of cyclin D1 and CDC25A, two pivotal mitogenic proteins overexpressed and positively regulated by constitutively active STAT3 in CRC (Carpenter and Lo, 2014). We also show that the addition of exogenous progranulin fails to rescue the negative effects of progranulin silencing on both cell proliferation and STAT3 function. Our data are in line with the work of Yeh and colleagues demonstrating a role for intracellular, but not extracellular progranulin, in sustaining STAT3 tyrosine phosphorylation and oncogenic activity in breast cancer cells (Yeh *et al*., 2015).

Time‐course analysis shows that block of cell cycle in progranulin‐deficient cells is followed by activation of caspase‐3 and induction of programmed cell death in association with the specific downregulation of STAT3‐related anti‐apoptotic proteins. Our data confirm and expand on previous studies showing aberrant levels of progranulin in different cancer types, such as hematologic, digestive, and gynecologic cancers (Arechavaleta‐Velasco *et al*., 2017), as well as the pro‐mitogenic and pro‐survival roles of this protein in malignant cells (Arechavaleta‐Velasco *et al*., 2017). The demonstration that progranulin knockdown sensitizes CRC cells to cytotoxic agents is in line with previous reports associating increased levels of progranulin with chemoresistance and a worse prognosis in hepatocellular carcinoma (Cheung *et al*., 2011; Wong *et al*., 2014), breast cancer (Abrhale *et al*., 2011; Kim and Serrero, 2006), multiple myeloma (Wang *et al*., 2006), and ovarian cancer (Pizarro *et al*., 2007). However, the mechanism by which progranulin promotes drug resistance in the above‐mentioned cancer is unknown. According to the recent discovery that STAT3 inhibitors may improve the outcome of chemotherapy in cancer patients (Yang *et al*., 2015; Zhao *et al*., 2016), it is tempting to speculate that the supporting role of progranulin on STAT3 activity might be the reason, or among the reasons, underlying such an effect. While this study was ongoing, Pan *et al*. documented upregulation of progranulin in CRC tissues and showed that, in DLD‐1 and HCT‐116 CRC cells, progranulin silencing with small interfering RNA decreases proliferation and increases apoptosis (Pan *et al*., 2018). In contrast with our results, the authors linked such effects with a G0/G1 phase cell cycle arrest and a modulation of the phospho‐MAPK/ERK pathway. The reasons for such a discrepancy remain unknown but could rely on differences in the experimental procedures and/or approaches used to downregulate progranulin expression (i.e., siRNA versus ASO) (Watts and Corey, 2012). To extend our observations to primary human cells, we next tested whether progranulin ASO could affect p‐STAT3 Tyr705 expression, as well as cell proliferation, both in CRC cells incubated with TIL‐derived supernatants and in human CRC explants. Notably, progranulin inhibition hampers STAT3 activation and cancer cell growth in both experimental systems. As STAT3 signaling plays a pivotal role in the regulation of the cancer stromal and immune cells of the tumor microenvironment (Yu *et al*., 2007), the possible use of progranulin ASO in the clinic should take into account issues related to the modulation of STAT3 in such cell compartments. However, in support of progranulin knockdown‐based strategies are findings indicating a role of ASO‐mediated STAT3 silencing in promoting immunosurveillance by both increasing the immunogenicity of cancer cells via cell‐autonomous pathways and favoring the reprogramming of the tumor microenvironment toward an immunostimulatory state (Hong *et al*., 2015). Moreover, as recent reports indicate a role for progranulin in boosting epithelial–mesenchymal transition, migration, and invasion of CRC cells (Ding *et al*., 2018; Zhao *et al*., 2018), approaches aimed at targeting progranulin could further benefit patients with advanced stages of the disease.

Further experimentation to address potential STAT3‐independent effects and/or drawbacks of progranulin ASO in preclinical models of both early and advanced CRC is however needed and currently ongoing.

## 5 Conclusions

In conclusion, this study demonstrates the contribution of progranulin in sustaining STAT3 oncogenic activity in CRC and suggests that progranulin inhibitors, either alone or in combination with conventional cytotoxic agents, can be promising approaches for therapeutic interventions in selected CRC subgroups marked by high levels of active STAT3.

## Conflict of interest

GM has received consultant honoraria from AbbVie. The other authors declare no conflict of interest.

## Author contributions

FL and FC performed most of the experiments, analyzed data, and contributed to write the manuscript; ADG, VD, DDF, AO, IS, and SS performed the experiments; IM contributed to collect human samples and critically revise the manuscript; NS and GM critically revised the manuscript; CS was responsible for the study concept and design, performed the experiments, analyzed data, and wrote the manuscript.

## Supporting information


**Fig. S1.** Progranulin RNA transcripts are increased in human CRC.
**Fig. S2.** Representative western blots showing progranulin expression in culture supernatants of HCEC‐1CT, HCT‐116 and HT‐29 cells.
**Fig. S3.** Immunofluorescence analysis by confocal microscopy of progranulin and p‐STAT3 Tyr705 in untreated HCT‐116 cells.
**Fig. S4.** Progranulin does not associate with JAK2 in HCT‐116 cells.
**Fig. S5.** Tumor necrosis factor (TNF)‐α, but not IL‐6, IL‐22 or IL‐17A, significantly enhances progranulin expression in HCEC‐1CT cells.
**Fig. S6.** Addition of exogenous progranulin does not rescue cell growth arrest and defects in STAT3 activity in progranulin antisense oligonucleotide (ASO)‐transfected HCT‐116 cells.
**Fig. S7.** Effect of chemotherapeutics on CRC cell survival.
