# Supplementary material for: Progranulin sustains STAT3 hyper‐activation and oncogenic function in colorectal cancer cells
Source: Mol Oncol. 2019 Aug 10;13(10):2142–59. doi: 10.1002/1878-0261.12552 (PMC6763778; doi:10.1002/1878-0261.12552)
Supplement: Supplementary file 1 — Fig. S1. Progranulin RNA transcripts are increased in human CRC. Fig. S2. Representative western blots showing progranulin expression in culture supernatants of HCEC‐1CT, HCT‐116 and HT‐29 cells. Fig. S3. Immunofluorescence analysis by confocal microscopy of progranulin and p‐STAT3 Tyr705 in untreated HCT‐116 cells. Fig. S4. Progranulin does not associate with JAK2 in HCT‐116 cells. Fig. S5. Tumor necrosis factor (TNF)‐α, but not IL‐6, IL‐22 or IL‐17A, significantly enhances progranulin expression in HCEC‐1CT cells. Fig. S6. Addition of exogenous progranulin does not rescue cell growth arrest and defects in STAT3 activity in progranulin antisense oligonucleotide (ASO)‐transfected HCT‐116 cells. Fig. S7. Effect of chemotherapeutics on CRC cell survival. [file MOL2-13-2142-s001.pdf]

Supplementary Figures

Progranulin sustains STAT3 hyper-activation and oncogenic function in colorectal cancer cells

Federica Laudisi, Fabio Cherubini, Antonio Di Grazia, Vincenzo Dinallo, Davide Di Fusco, Eleonora Franzè, Angela Ortenzi, Illari Salvatori, Silvia Scaricamazza, Ivan Monteleone, Naoya Sakamoto, Giovanni Monteleone and Carmine Stolfi

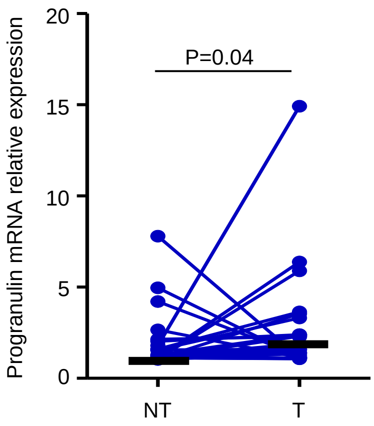

Supplementary Figure 1: Progranulin RNA transcripts are increased in human CRC. Paired colonic explants taken from nontumoral (NT) and tumoral (T) areas of 26 patients with sporadic CRC were analyzed for progranulin expression by real-time PCR. Levels were normalized to β-actin. Each point represents the value of progranulin mRNA in a single patient. Horizontal bars indicate median value. Differences among groups were compared using the Mann-Whitney U test.

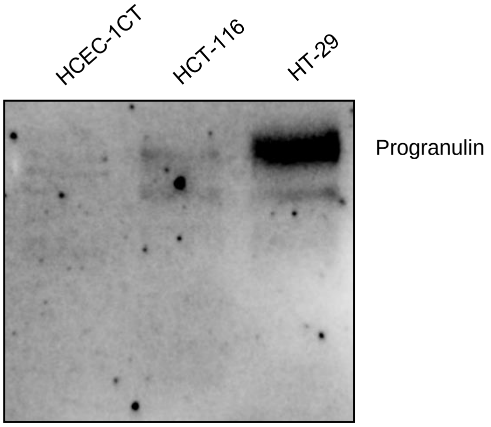

Supplementary Figure 2: Representative western blots showing progranulin expression in culture supernatants of HCEC-1CT, HCT-116 and HT-29 cells. One of three representative experiments in which similar results were obtained is shown.

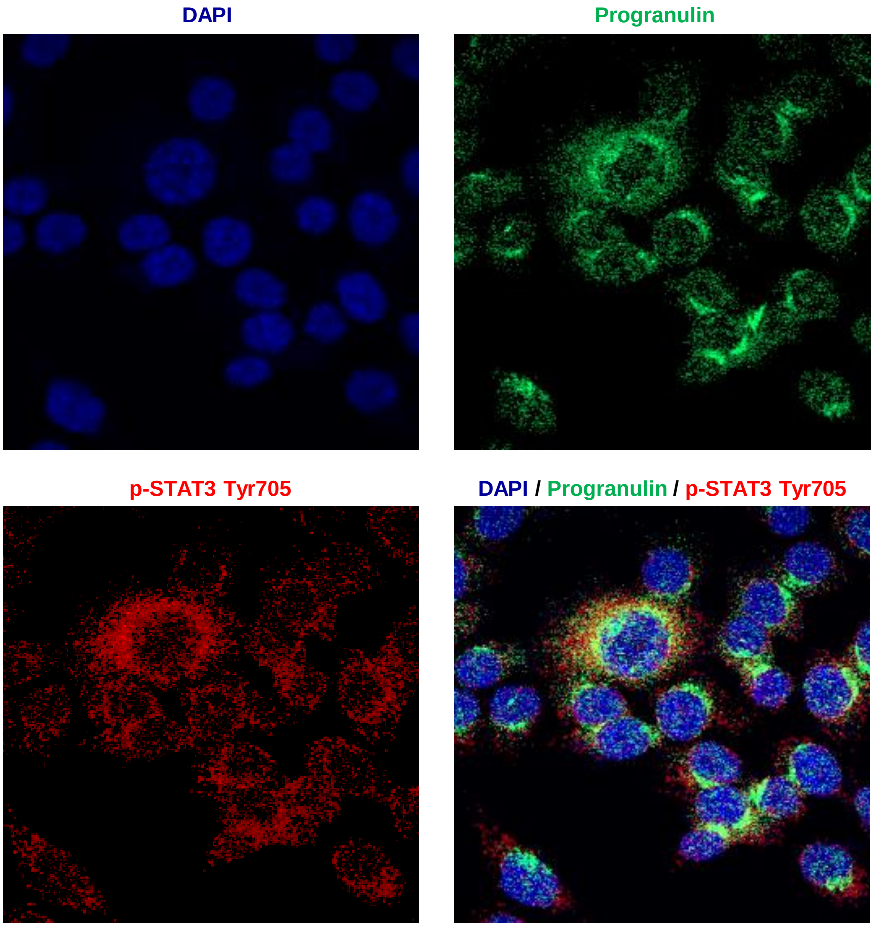

Supplementary Figure 3: Immunofluorescence analysis by confocal microscopy of progranulin and p-STAT3 Tyr705 in untreated HCT-116 cells. Progranulin and p-STAT3 Tyr705 are labeled in green and red respectively. Nuclei are stained with 4’,6-diamidino-2-phenylindole (DAPI) (blue) (original magnification x63). The figure is representative of three separate experiments in which similar results were obtained.

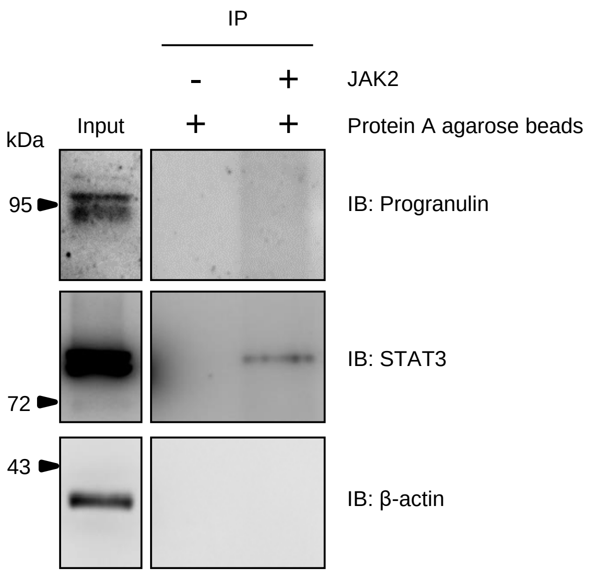

Supplementary Figure 4: Progranulin does not associate with JAK2 in HCT-116 cells. Total proteins extracted from HCT-116 cells were immunoprecipitated by an anti-human JAK2 antibody and then subjected to immunoblotting analysis using progranulin, STAT3 and β-actin antibodies. One of three representative experiments in which similar results were obtained is shown.

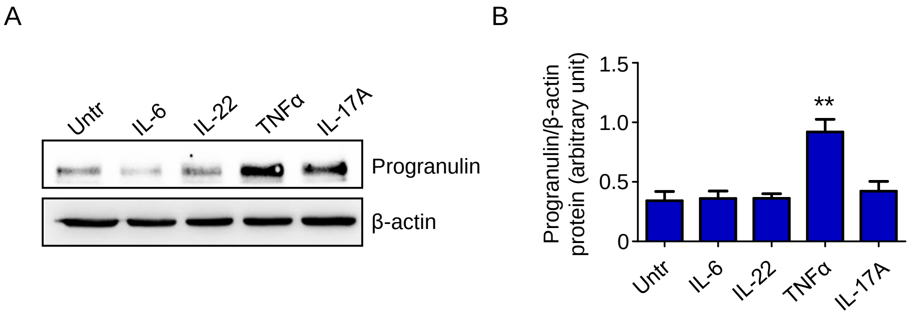

Supplementary Figure 5: Tumor necrosis factor (TNF)-α, but not IL-6, IL-22 or IL-17A, significantly enhances progranulin expression in HCEC-1CT cells. (A) Representative western blotting showing progranulin expression in HCEC-1CT cells stimulated or not with IL-6, IL-22, TNF-α and IL-17A (all used at 25 ng/ml) for 24 hours. β-actin was used as a loading control. One of three representative experiments in which similar results were obtained is shown. (B) Quantitative analysis of progranulin/β-actin protein ratio in total extracts of HCEC-1CT cells stimulated as indicated in A, as measured by densitometry scanning of western blots. Values are expressed in arbitrary units and are the mean ± SEM of three experiments. Differences among groups were compared using one-way analysis of variance (ANOVA) followed by Tukey's post hoc test. TNF-α-stimulated cells vs untreated cells, ***P* < 0.01.

A

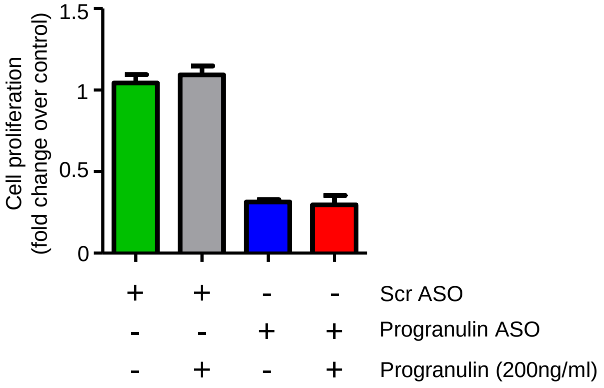

B

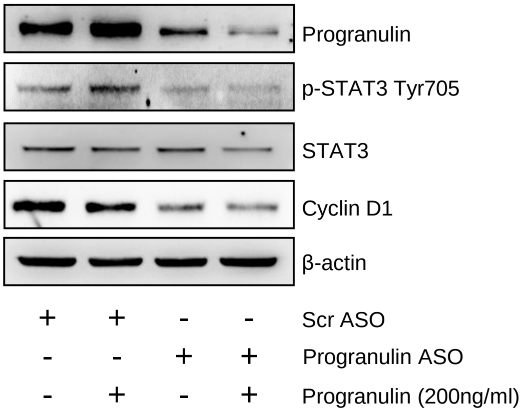

Supplementary Figure 6: Addition of exogenous progranulin does not rescue cell growth arrest and defects in STAT3 activity in progranulin antisense oligonucleotide (ASO)-transfected HCT-116 cells. (A) HCT-116 were transfected with either scrambled (Scr) or progranulin ASO (both used at 200 nM). After 24 hours cells were washed with PBS and cultured with or without human recombinant progranulin for further 24 hours. Cell proliferation was assessed by 5-bromodeoxyuridine (BrdU) proliferation assay kit. Data indicate mean ± SEM of three experiments. (B) Total proteins from HCT-116 cells treated as indicated in A were extracted and evaluated for progranulin, p-STAT3 Tyr705, STAT3, and Cyclin D1 expression by western blotting. β-actin was used as loading control. One of three representative experiments where similar results were obtained is shown.

A

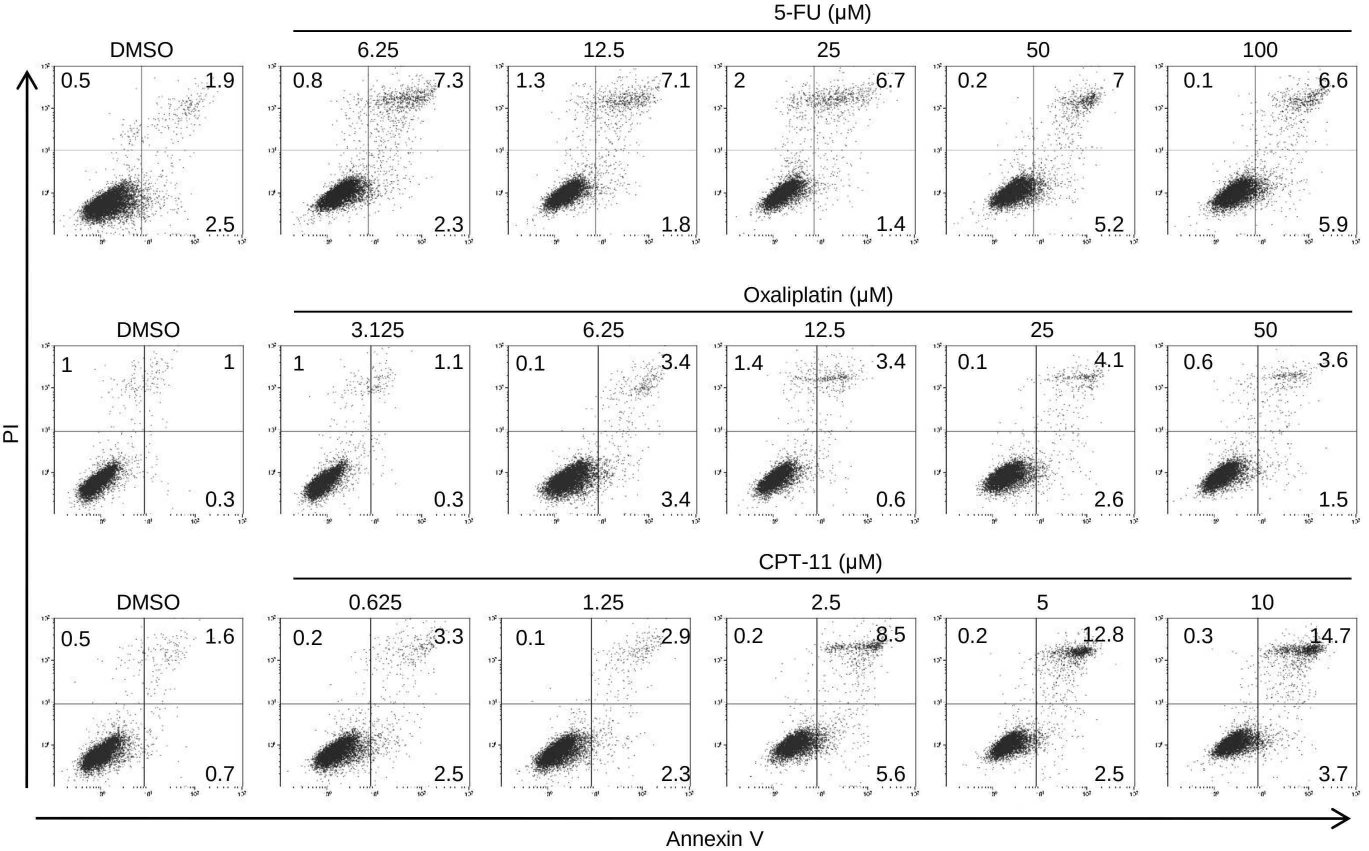
5-FU (μM)
DMSO 6.25 12.5 25 50 100
0.5 1.9 2.5
0.8 7.3 2.3
1.3 7.1 1.8
2 6.7 1.4
0.2 7 5.2
0.1 6.6 5.9
Oxaliplatin (μM)
DMSO 3.125 6.25 12.5 25 50
1 1 0.3
1 1.1 0.3
0.1 3.4 3.4
1.4 3.4 0.6
0.1 4.1 2.6
0.6 3.6 1.5
CPT-11 (μM)
DMSO 0.625 1.25 2.5 5 10
0.5 1.6 0.7
0.2 3.3 2.5
0.1 2.9 2.3
0.2 8.5 5.6
0.2 12.8 2.5
0.3 14.7 3.7
PI
Annexin V

B

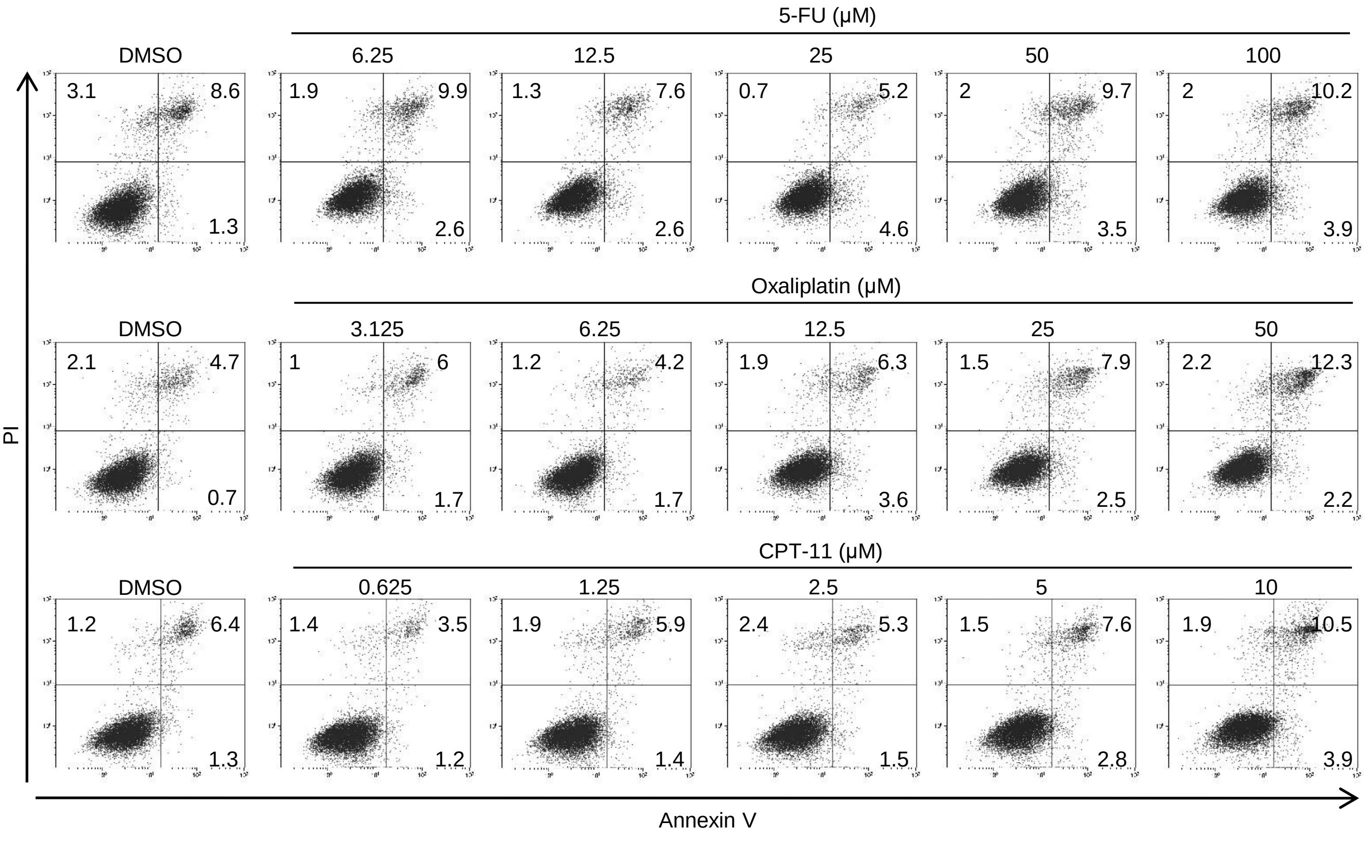
5-FU (μM)
DMSO 6.25 12.5 25 50 100
3.1 8.6 1.3
1.9 9.9 2.6
1.3 7.6 2.6
0.7 5.2 4.6
2 9.7 3.5
2 10.2 3.9
Oxaliplatin (μM)
DMSO 3.125 6.25 12.5 25 50
2.1 4.7 0.7
1 6 1.7
1.2 4.2 1.7
1.9 6.3 3.6
1.5 7.9 2.5
2.2 12.3 2.2
CPT-11 (μM)
DMSO 0.625 1.25 2.5 5 10
1.2 6.4 1.3
1.4 3.5 1.2
1.9 5.9 1.4
2.4 5.3 1.5
1.5 7.6 2.8
1.9 10.5 3.9
PI
Annexin V

Supplementary Figure 7: Effect of chemotherapeutics on CRC cell survival. Representative dot-plots showing the percentage of Annexin V (AV)- and/or propidium iodide (PI)-positive HCT-116 (A) and HT-29 cells (B) treated with DMSO (vehicle) or increasing doses of 5-fluorouracil (5-FU), oxaliplatin and camptothecin-11 (CPT-11) as indicated for 48 and 60 hours respectively. Numbers indicate the percentage of AV and/or PI-positive cells within the designated areas. One of 3 representative experiments in which similar results were obtained is shown.
